# EHMTI-0241. Association between migraine and sod1 and sod2 genes polymorphisms: the biobim study

**DOI:** 10.1186/1129-2377-15-S1-B4

**Published:** 2014-09-18

**Authors:** P Barbanti, R Palmirotta, ML De Marchis, G Ludovici, C Ialongo, G Egeo, C Aurilia, L Fofi, P Ferroni, D Della Morte, F Guadagni

**Affiliations:** 1 Headache and Pain Unit Department of Neurological Motor and Sensorial Sciences, IRCCS San Raffaele, Rome, Italy; 2 Interinstitutional Multidisciplinary Biobank (BioBIM) Biomarker Discovery and Advanced Technologies (BioDAT), IRCCS San Raffaele, Rome, Italy; 3 Department of Internal Medicine, University of Rome "Tor Vergata", Rome, Italy; 4 Department of System Medicine, University of Rome "Tor Vergata", Rome, Italy

## Introduction

Several studies suggest that an increased oxidative stress is a key event in migraine, especially in the form with aura. One of the most efficient first line antioxidant defense system of cells exposed to oxygen is Superoxide Dismutase (SOD) system. The SOD1 gene presents a polymorphism (A/C substitution - rs2234694) resulting in lower serum activitiy in subjects carrying CC genotype. SOD2 gene presents a polymorphism (C/T transition -rs4880 – Ala16Val) which leads to an instable mRNA and reduced transport of the enzyme in TT genotype carriers.

## Aim

To investigate whether these variants are associated to migraine without aura (MO), migraine with aura (MA), or chronic migraine (CM) in a cohort of Caucasian patients from the Interinstitutional Multidisciplinary BioBank (BioBIM).

## Methods

From 2009 to 2013, 490 Caucasian unrelated migraineurs and 246 healthy control subjects were consecutively recruited at the our Headache and Pain Unit. Demographic and clinical migraine features were carefully detailed and gathered with face-to face interviews.The polymorphisms were determined by direct sequencing analysis.

## Results

Genotypes and the allele frequencies of polymorphisms in migraineurs and controls did not significantly differ from those predicted by the Hardy–Weinberg equilibrium. When considering unilateral cranial autonomic symptoms (UAs), we observed a significant increase of SOD2 rs4880 TT (Val) genotype in MA (P=0.042).

## Conclusions

SOD2 TT genotype correlates with MA with UAs. We hypothesize that in SOD2 TT carriers with MA, a defective control of oxidative phenomena characterizing cortical spreading depression leads to a more intense antidromic stimulation of trigeminal endings, thus triggering the trigemino-autonomic reflex.

No conflict of interest.

